# Early Versus Delayed Thoracic Endovascular Aortic Repair for Blunt Traumatic Aortic Injury: A Systematic Review and Meta-Analysis

**DOI:** 10.7759/cureus.41078

**Published:** 2023-06-28

**Authors:** Pranathi Rudra, Rayner Cardoso, Sophia Echevarria, Berfin Kaya, Ramal Abdullah, Rishabh Baskara Salian, Shah Zaib Bhindar, Annu Zerin, Tirath Patel, Zain Abdin, Mohammed Al-Tawil

**Affiliations:** 1 Internal Medicine, Gandhi Medical College, Secunderabad, IND; 2 Medical School, All India Institue of Medical Sciences, Jodhpur, IND; 3 General Surgery, Universidad Mayor de San Simon, Cochabamba, BOL; 4 Obstetrics and Gynaecology, Faculty of Medicine, Izmir Kâtip Celebi University, Izmir, TUR; 5 Medical School, Foundation University Medical College, Foundation University School of Health Sciences (FUSH), Islamabad, PAK; 6 Medical School, Kasturba Medical College, Mangalore, IND; 7 Orthopaedic Surgery, Ghurki Trust and Teaching Hospital, Lahore, PAK; 8 Internal Medicine, All India Institute of Medical Sciences, Bhubaneswar, IND; 9 Medical School, American University of Antigua, St. John's, ATG; 10 Critical Care Medicine, IMG Helping Hands, Albuquerque, USA; 11 Surgery, Al-Quds University, Jerusalem, PSE

**Keywords:** btai, trauma, blunt aortic injury, tevar, thoracic endovascular aortic repair

## Abstract

Blunt aortic injury is the second most prevalent cause of patient fatalities post-trauma, closely following head injuries as the leading cause. In recent years, thoracic endovascular aortic repair (TEVAR) has evidently improved survival rates and reduced complications in patients suffering from blunt traumatic aortic injury (BTAI) in comparison to open surgery and non-operative management.

It is difficult to characterize the appropriate criteria for the timing of TEVAR, whether early or delayed for BTAI, considering the discrepancies related to timing.

Electronic databases, including PubMed, Scopus, the Cochrane Central Register of Controlled Trials (CENTRAL), and Embase, were searched through April 2023. The primary outcomes were short-term mortality and hospital and intensive care unit (ICU) stays. Time to TEVAR, acute respiratory distress syndrome, sepsis, deep vein thrombosis, delayed stroke, and renal failure were also evaluated.

We included a total of seven studies, comprising 4177 patients who met the inclusion criteria. Short-term mortality was significantly higher in the early TEVAR group (RR: 1.86; 95% confidence interval (CI); (1.26-2.74); p<0.001; I^2^=33%). In contrast, the ICU length of stay was significantly shorter in the early group (mean difference: -2.82 days; 95% CI; (-4.09 - -1.56); p<0.0001; I^2^=55%). There was no significant difference between both groups in the presenting profile or postoperative complications.

Patients undergoing delayed TEVAR had markedly lower mortality rates but a longer ICU stay. The need for future studies with more robust designs is imperative to investigate the factors influencing the timing of repair and the associated outcomes.

## Introduction and background

Among all traumatic injuries, one of the most time-sensitive is blunt traumatic aortic injury (BTAI). It is the second most prevalent cause of patient fatalities, closely following head injuries as the leading cause [1]. It has been reported that as many as 80% of patients with BTAI die before being admitted to the hospital, and among those who survive, the in-hospital death rate can reach up to 46%. Rapid deceleration from vehicle collisions or falls from great heights can cause a potentially fatal BTAI [2]. To effectively manage BTAI, the Society for Vascular Surgery (SVS) grading system has been widely adopted by vascular surgeons. This grading system categorizes BTAI into four distinct grades: I (intimal tears), II (intramural hematoma), III (pseudoaneurysm), and IV (rupture) [3]. Traditional treatment approaches for aortic injuries involved open surgical repair, which often presented significant challenges and risks.

Thoracic endovascular aortic repair (TEVAR) has revolutionized the treatment of aortic injuries. In recent years, TEVAR has shown promising results in terms of improved survival rates and reduced complications compared to traditional approaches. It has become the mainstay of treatment for BTAI [3-4] and is increasingly advocated over medical therapy even in stable aortic pathologies [5-7].

Furthermore, it has been linked to superior outcomes in terms of reduced neurological and vascular complications in comparison to open surgery and non-operative management [8]. Despite the growing consensus regarding the superiority of TEVAR, the optimal timing for intervention remains a topic of debate. Guidelines established by the SVS recommend early TEVAR for BTAI patients, with delayed TEVAR reserved for patients at high risk who have severe injuries or comorbidities [9]. Some reports [10] found no appreciable changes in patient outcomes between early and delayed TEVAR, while others reported significantly improved outcomes with delayed TEVAR [11]. These discrepancies have made it challenging to establish definitive criteria for the use of delayed TEVAR in BTAI cases and to determine its overall impact on patient survival. In light of these results, it is difficult to characterize the criteria for using delayed TEVAR for BTAI, and its overall effects on patient survival are still unclear.

Considering such timing discrepancies, we conducted a systematic review of the available literature to investigate the optimal timing of TEVAR for patients presenting with BTAI.

Materials and methods

Literature Search

Search terms were formulated using the Population, Intervention, Comparator, and Outcome (PICO) framework to identify papers comparing early versus delayed BTAI. The databases searched were PubMed, Scopus, the Cochrane Central Register of Controlled Trials (CENTRAL), and Embase through April 2023. The updated version of the Preferred Reporting Items for Systematic Reviews and Meta-Analyses (PRISMA) statement and recommended guidelines were used to guide such a process [12-13].

The terms used were "blunt" OR "aortic injury" OR "traumatic" OR "trauma" OR "BTAI" OR "TAI" OR "TTAL" OR "BAAI" AND "endovascular repair" OR "endovascular aortic repair" OR "endovascular surgery" OR "TEVAR" AND "early" OR "delayed" OR "late" OR "urgent" OR "emergent" OR "timing" OR "24 hours". The identified studies were screened after reading the abstracts of the studies. Duplicates were also omitted from the total number of titles and abstracts accumulated. All the studies selected for this analysis were chosen after applying the inclusion and exclusion criteria through the different screening stages.

Inclusion and Exclusion Criteria

The main inclusion criteria were studies that compared the outcomes between the early and delayed intervention of TEVAR in blunt traumatic thoracic aortic injuries. The included studies have to define the number of patients in both groups and report at least one comparative outcome of interest. If the studies did not include such comparative outcomes, they were excluded from the analysis. To be included, studies had to: 1) be clinical trials or observational cohort studies; 2) contain more than 10 patients; and 3) be studies with patients above 16 years old exposed to blunt thoracic or abdominal aortic trauma injuries that were intervened on with an early (<24 hours) or delayed (>24 hours) TEVAR.

Exclusion criteria were: 1) systematic review articles, meta-analyses, review articles, case reports, editorials, study protocols, abstracts, commentaries, or letters; 2) patients under 16 years of age; 3) adult patients with open surgery intervention; 4) other causes of aortic disease like aneurysms and dissections; or 5) non-English studies.

**Figure 1:**
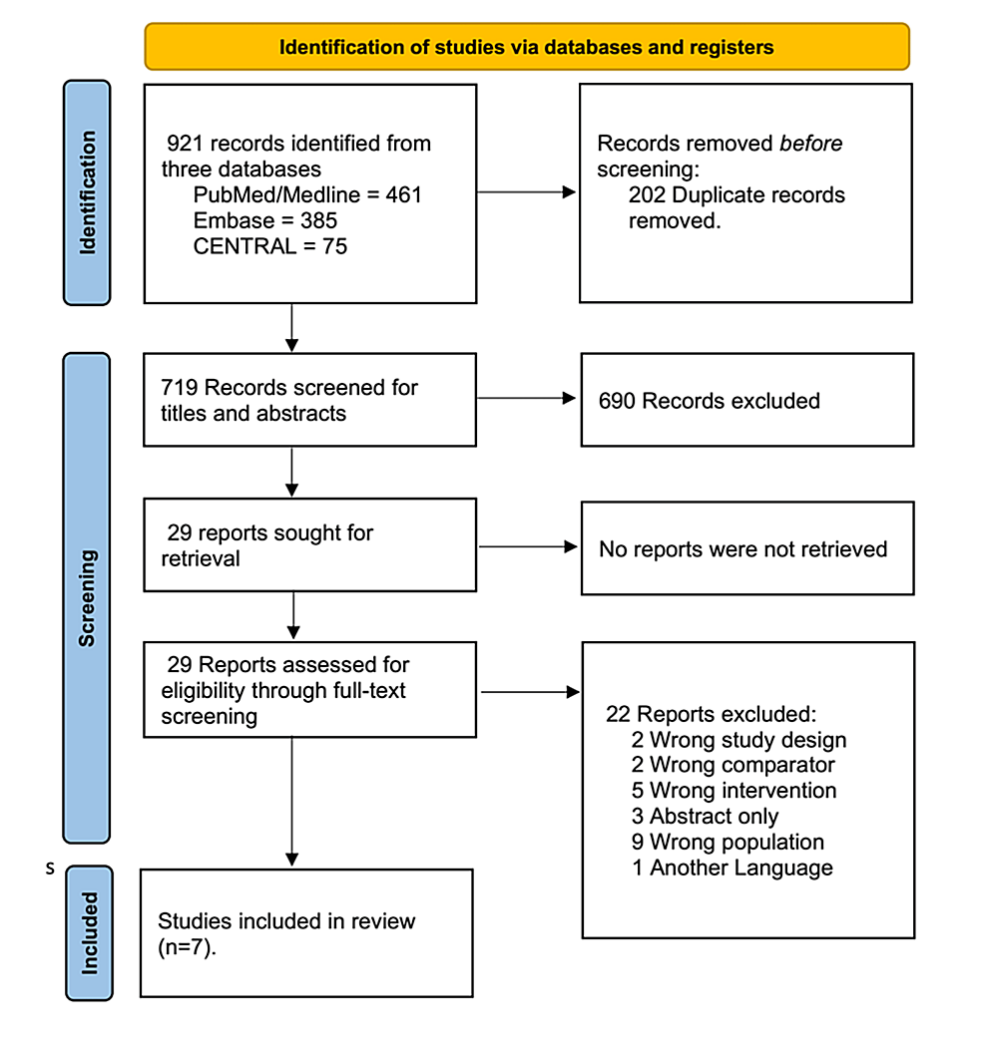
The PRISMA flow diagram illustrates the different stages of study selection PRISMA: Preferred Reporting Items for Systematic Reviews and Meta-Analyses

At both the abstract and full-text review stages, at least two independent reviewers examined each study, and a third reviewer resolved any conflicts. Figure 1 shows the PRISMA flowchart process of filtering from the original literature search to the final studies that are included in this meta-analysis.

Data Extraction, Outcomes, and Quality Assessment

The cumulative data were extracted from each included article. Two independent investigators extracted data from each record. This was further revised by two other members, and the results were compared to resolve any conflicts by consensus, if present. The included studies' summaries were extracted. Baseline parameters, including patients' age, gender, Injury Severity Score (ISS), BTAI grade, and concomitant injuries, were extracted.

The primary endpoint for our analysis was early mortality. Early mortality was defined as any death recorded in the study within 30 days of the procedure. Secondary endpoints included in-hospital and intensive care unit (ICU) length of stay, sepsis, delayed stroke, acute respiratory distress syndrome (ARDS), deep vein thrombosis (DVT), and renal failure.

Categorical data were extracted as events and totals for each group, while continuous data were coded as mean and standard deviations. If the data were reported in other formats, the method by Wan and colleagues [13] was used to perform the necessary conversion.

We used the Newcastle-Ottawa (NOS) risk of bias assessment tool to assess the quality of the included studies. Two reviewers assessed the risk of bias independently, and the final table was assembled based on their agreement. The assessment cut-off for follow-up length was set at 30 days. We regarded follow-up as sufficient if no more than 10% of the cohort had lost follow-up.

Statistical Analysis

This meta-analysis was conducted in line with the recommendations from the Cochrane Collaboration and Meta-analysis of Observational Studies in Epidemiology [14-15]. We used the Review Manager software, version 5.4.1 (Cochrane Foundation), for statistical analysis. The Mantel-Haenszel random-effects models were used to estimate the risk ratio (RR) and the corresponding 95% confidence intervals (CIs) for binary outcomes. To estimate the weighted mean difference for continuous outcomes, we used the inverse variance method. The Q-test for heterogeneity (Cochran 1954) and the I² statistics were used to assess statistical heterogeneity; I² >50% indicated substantial heterogeneity. A p-value of less than 0.05 was considered a statistically significant outcome. A sensitivity analysis was conducted to test the robustness of the results, and the results were reported. Publication bias was initially assessed by visual inspection of the funnel plot and confirmed by Egger’s regression tests.

## Review

Results

Included Studies and Population Characteristics

During the initial search, we identified 921 records for eligibility screening; 29 full-text studies were assessed for eligibility; and seven studies were eligible and included in our meta-analysis (Figure 1).

A total of 4177 patients were included in our analysis, of whom 2966 (71%) underwent early TEVAR and 1211 (29%) underwent delayed TEVAR for BTAI repair. Only two studies reported the grade of BTAI, while another single study included only patients with grade III BTAI "pseudoaneurysm". Additional characteristics of the included studies and patient baseline data are shown in Tables 1-4. The included studies were of moderate to good quality.

**Table 1:** Characteristics and conclusions of the included studies

Study name and year	Study period	Study design	Total no. of patients		Study conclusion
			Early	Delayed	
Alarhayem et al. (2021) [4]	2012-2017	Retrospective cohort study	2118	703	Delayed TEVAR for BTAI beyond 24 hours improves survival compared to early repair, despite similar injury characteristics.
Arbabi et al. (2021) [10]	2016-2020	Prospective cohort study	77	23	The timing of TEVAR for patients with concurrent BTAI and TBI does not affect stroke, mortality, or aortic-related outcomes, emphasizing the influence of patient factors in guiding intervention.
Botta et al. (2008) [16]	1998-2007	Retrospective cohort study	11	16	Similar outcomes were observed in both the early and delayed groups. When clinical conditions enable it, TEVAR can be safely delayed to manage the major associated lesions.
Demetriades et al. (2009) [11]	2005-2007	Prospective cohort study	109	69	Delayed repair of stable BTAI improves survival regardless of associated injuries, but it is linked to longer ICU stays and a higher complication rate in patients without major associated injuries.
Marcaccio et al. (2018) [17]	2009-2013	Retrospective cohort study	378	129	Delayed TEVAR was associated with lower mortality rates in patients with BTAI, highlighting the need to re-evaluate existing guidelines for BTAI management.
Romijn et al. (2023) [18]	2016-2019	Propensity-score matched study	1054	285	In this propensity-score-matched study, delayed TEVAR was associated with lower mortality risk, even after adjusting for aortic injury grade.
Smeds et al. (2016) [19]	2004-2012	Retrospective cohort study	10	13	Watchful waiting may be permissible in patients with Grade III BAI and other associated multisystem trauma. This allows for repairs in a more controlled environment.

**Table 2:** Baseline characteristics of the patients

Study name and year	Number of patients		Age (Mean ± SD)		Male sex (%)		Injury Severity Score (ISS) (Mean ± SD)	
	Early	Delayed	Early	Delayed	Early	Delayed	Early	Delayed
Alarhayem et al. (2021) [4]	2118	703	42.1 ± 20.2	43.2 ± 20.1	74.8%	75.2%	32.8 ± 13.3	30.4 ± 13.9
Arbabi et al. (2021) [10]	77	23	39 ± 21.91	48 ± 23.71	80.5%	73.9%	41 ± 4.1	38 ± 3.8
Botta et al. (2008) [16]	11	16	38.5 ± 12.3	35.8 ± 10.2	81.81%	93.75%	-	-
Demetriades et al. (2009) [11]	109	69	39.1 ± 17.7	39.9 ± 19.1	74.3%	81.2%	38.2 ± 10.6	40.9 ± 12.6
Marcaccio et al. (2018) [17]	378	129	40.75 ± 4.94	42 ± 6.18	75.4%	74.4%	33.7 ± 2.5	33.2 ± 2.8
Romijn et al. (2023) [18]	1054	285	40.25 ± 5.60	43 ± 4.56	75.05%	71.23%	-	-
Smeds et al. (2016) [19]	10	13	45.5	42.4	80%	69%	38 ± 13.2	41 ± 11.0

**Table 3:** Summary of aortic injury grades from reporting studies

Study name and year	Grade I		Grade II		Grade III		Grade IV	
	Early	Delayed	Early	Delayed	Early	Delayed	Early	Delayed
Arbabi et al. (2021) [10]	1.3%	8.7%	10.4%	8.7%	77.9%	78.3%	10.4%	4.3%
Romijn et al. (2023) [18]	28%	47%	24%	30%	49%	23%	0%	0%
Smeds et al. (2016) [19]	0%	0%	0%	0%	100%	100%	0%	0%

**Table 4:** Summary of common concomitant injuries reported

Study name and year	Rib fracture		Splenic injury		Neurological injury		Cervical spine fracture	
	Early	Delayed	Early	Delayed	Early	Delayed	Early	Delayed
Arbabi et al. (2021) [10]	50.6%	34.8%	29.9%	34.8%	-	-	33.8%	17.4%
Botta et al. (2008) [16]	-	-	-	-	-	-	-	-
Demetriades et al. (2009) [11]	-	-	-	-	14%	23.2%	-	-
Marcaccio et al. (2018) [17]	69.8%	73.6%	25.7%	26.4%	22.5%	38.8%	-	-
Romijn et al. (2023) [18]	67%	70%	28%	30%	2.5%	1.1%	-	-
Smeds et al. (2016) [19]	-	-	-	-	50%	38%	-	-

Primary Outcome

**Figure 2:**
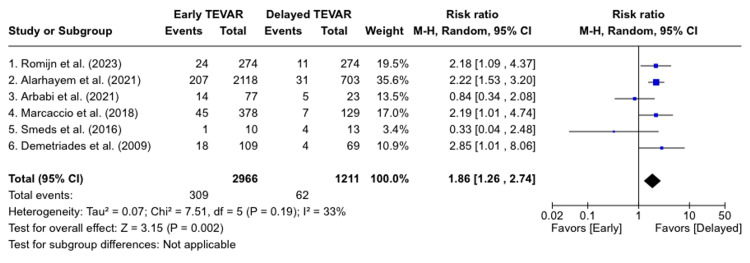
Short-term mortality following TEVAR contrasted between both groups M-H: Mantel Haenszel model [4,10,11,17-19]

All included studies reported short-term mortality, which was significantly higher in the early TEVAR group (RR: 1.86; 95% confidence interval (CI); (1.26-2.74); p<0.001; the proportion of the variance in the observed effect (I2) =33%). Figure 2 shows the short-term mortality following TEVAR contrasted between both groups [4,10,11,17-19].

**Figure 3:**
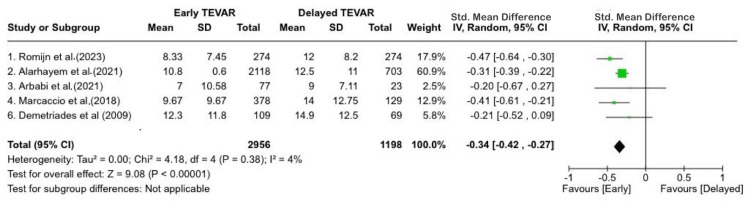
ICU length of stay contrasted between both groups. IV: inverse-variance [4,10,11,17-18].

There was no significant change to the results when sensitivity analysis was performed. However, the ICU length of stay was significantly shorter in the early group (mean difference: -2.82 days), 95%CI; (-4.09 - -1.56); p<0.0001; I2=55%). Figure 3 displays the length of stay in the intensive care unit (ICU) contrasted between both groups [4,10,11,17-18]. We could not estimate differences regarding in-hospital stays due to severe heterogeneity between studies and likely different definitions of the starting timepoint. No publication bias was detected.

Secondary Outcomes

Secondary outcomes were reported in four studies. There was no significant difference between early and late TEVAR groups in terms of post-procedural complications, including delayed stroke (RR: 1.08; 95% CI; (0.64-1.83); p=0.77; I2=0%), deep vein thrombosis (RR: 0.92; 95% CI; (0.30-2.83); p=0.89; I2=68%), renal failure (RR: 0.86; 95% CI; (0.24-3.07) p=0.82; I2=37;), or sepsis (RR: 0.75; 95% CI; (0.53-1.07); p=0.59; I2=0%) following endovascular repair. We could not estimate differences regarding time-to-TEVAR due to the severe heterogeneity between the included studies.

Table 5 shows the quality assessment of the included studies.

**Table 5:** Risk of bias assessment using the Newcastle-Ottawa Scale (in-hospital follow-up was accepted as an appropriate follow-up length; adequacy of follow-up meant less than 10% loss) The Newcastle-Ottawa Scale (NOS) is a tool used to assess the quality of non-randomized studies, particularly in epidemiology and systematic reviews. The scale aims to evaluate the risk of bias in observational studies, including case-control and cohort studies. The NOS assigns stars and zeros to specific criteria within three main categories: selection of study groups, comparability of groups, and ascertainment of exposure or outcome. A higher total score indicates a lower risk of bias and higher study quality. Please refer to the table for a detailed breakdown of the criteria and corresponding star or zero scoring.

Study name and year	Selection				Comparability		Outcome			Score
	Representing the Exposed	Selection of Non-Exposed	Ascertainment of Exposure	Outcome of interest not present at the start of the study	Main factor	Additional factor	Assessment	Follow-up length	Adequacy of follow-up	
Romijn et al. (2023) [18]	★	★	★	★	★	★	★	★	★	9
Alarhayem et al. (2021) [4]	★	★	★	★	★	0	★	0	★	6
Arbabi et al. (2021) [10]	★	★	★	★	★	0	★	★	★	7
Marcacio et al. (2018) [17]	★	★	★	★	★	0	★	★	★	7
Smeds et al. (2016) [19]	★	★	★	★	★	0	★	★	★	7
Demetriades et al. (2009) [11]	★	★	★	★	★	0	★	0	★	6
Botta et al. (2008) [16]	★	★	0	★	★	0	★	0	★	5

Discussion

It was earlier thought that early TEVAR would have a better outcome as compared to delayed TEVAR due to the higher risk of complications. In this systemic review and meta-analysis, 4177 patients who underwent TEVAR for BTAI from seven studies were included in the analysis. These studies included both prospective studies and retrospective analyses. Among the patients, 2966 (71%) underwent early repair (<24 hours) for BTAI, while 1211 (29%) underwent delayed repair (>24 hours). The primary conclusions drawn from the study are as follows: 1) in the delayed repair group, the short-term mortality rate was significantly lower; 2) the ICU length of stay was significantly lower in the early group [4,10,11,17-18]. No significant differences were noted in terms of preoperative characteristics or postoperative complications. In terms of reported data across the studies, there were no discernible trends observed, and the patients had similar baseline characteristics and presentation data.

TEVAR has recently emerged as a treatment option for patients with BTAI and is considered a favorable alternative to open surgery. Notably, there were no previous meta-analyses on this subject at the time this study was started.

The definition of early versus delayed repair in BTAI can vary among studies, with no universally accepted cut-off. Generally, delayed repair refers to interventions performed more than 24 hours after the initial injury, although different studies may use different time intervals. The 24-hour cut-off is commonly used as a clinical recommendation to avoid detrimental incidents such as aortic rupture. The seven-day cut-off was also used to define delayed repair in BTAI. This timeframe allows for assessing outcomes in patients who did not undergo immediate intervention. It acknowledges that certain patients may benefit from a delayed approach due to associated injuries and patient stability [20].

Blunt aortic injuries rarely occur in isolation, as patients often experience other vascular traumas [19]. A study by Smeds and colleagues [19] showed that despite similar ISS and Glasgow Coma Scale (GCS) scores upon arrival, patients in the delayed group had a higher incidence of additional trauma diagnoses compared to the immediate repair group. This highlights that undergoing early repair might necessarily lead to missing some other ‘possibly life-threatening’ injuries that contributed to the increased mortality in the early group.

The management of blood pressure and associated injuries plays a significant role in determining patient outcomes in BTAI. The use of antihypertensive and rate-controlling agents during the delay period has become standard practice to prevent further extension of the injury [21]. Endovascular repair with aortic stent grafts has shown reduced morbidity and mortality, particularly in stable patients treated with antihypertensives and β-blockade [21]. Moreover, advancements in noninvasive imaging techniques may aid in reducing the need for additional invasive procedures, further improving patient outcomes [21]. Moreover, investigations into other aortic pathologies have also underscored the significance of the timing of repair. For instance, in the context of uncomplicated type B aortic dissection, the timing of the repair has emerged as a crucial factor influencing patient outcomes [22]. These findings emphasize the importance of considering the optimal timing for intervention in different pathological conditions to achieve the best possible clinical results.

The decreased risk of spontaneous rupture with antihypertensives is a significant factor in determining outcomes. Delayed treatment was shown to allow for intramural hematoma regression, resulting in more stable landing zones for TEVAR, which subsequently led to higher success and lower complication rates as well as greater flexibility in terms of operative timing [23].

The evolving treatment strategies for BTAI have witnessed a shift from open surgical repair (OAR) to TEVAR and non-operative management (NOM). TEVAR is recommended for grades 2 to 4 injuries, while NOM is considered a reasonable strategy for mild injuries (grades 1 and 2). Grades 3 and 4 injuries typically require definitive repair with TEVAR or OAR, depending on the presence of comorbid injuries [9, 24]. A study by McCurdy et al. supports the recommendation for immediate medical correction followed by delayed repair, as delayed TEVAR was associated with better outcomes [24]. This approach allows for the stabilization of the patient's condition before the intervention, reducing the risk of complications and improving the overall prognosis.

There is an emerging role for preprocedural anticoagulation. In a study by Zambetti et al. [25], delayed TEVAR, accompanied by procedural heparinization, proved to be a safe treatment approach irrespective of TBI, leading to improved survival rates and no adverse effects on neurologic function at discharge [25]. The authors also highlighted that the best cut-off timing for TEVAR intervention was identified as 14.8 hours in the study, as TEVAR mortality was higher before this time frame.

Certain risk factors for early mortality have been identified that assist in appropriate patient selection and risk stratification. These factors include increased age, elevated creatinine levels (indicative of acute renal failure), male gender, Injury Severity Scores reflecting the presence of non-aortic injuries, and involvement of the left subclavian artery [26]. Surprisingly, this study by Mohapatra et al. showed that other aortic-specific factors, such as the grade of aortic injury, did not emerge as predictors of mortality [26]. This observation suggests that considering the overall clinical presentation of the patient's trauma complex holds greater relevance in patient selection than focusing solely on the specific characteristics of the aortic injury itself.

Overall, the option of delayed repair for TAI patients with significant associated injuries is viable, given that blood pressure is adequately managed. This allows for improved resuscitation, patient stabilization, and the identification and management of life-threatening injuries. Still, such evidence needs to be tested in studies of higher quality and prospective nature, allowing for a further in-depth understanding of other factors that moderate outcomes in this population.

Limitations

The inclusion of a retrospective, observational study design in this meta-analysis introduces certain limitations. The absence of randomized clinical trials comparing delayed and early repairs diminishes the strength of the evidence. Synthesizing data solely from retrospective observational studies introduces potential biases and confounding factors that may affect the accuracy and reliability of the findings. Furthermore, there was a notable variability in the sample sizes among the included studies, which may impact the statistical power and accuracy of the results. Consequently, the generalizability of the findings to a larger group of high-risk patients for repeat surgical intervention may be limited. It is important to acknowledge that the decision for patients to undergo TEVAR via early or delayed repair was made independently by each surgical team, based on surgeon experience, judgment, and consideration of the extent of the aortic injury. This introduces selection bias, as factors not accounted for in the analysis may influence the choice of repair timing. Additionally, only two studies reported the grade of BTAI associated with the injury, which could have served as an important confounder in the decision of early versus delayed TEVAR. Recognizing and addressing these limitations is crucial when interpreting the findings of our study. Also, future studies in this area would benefit from prospective, randomized controlled trials with larger sample sizes and individual patient data analysis to enhance the strength and validity of results.

## Conclusions

Patients undergoing delayed TEVAR had markedly lower mortality rates but a longer ICU stay. The need for future studies with more robust designs is imperative to investigate the factors influencing the timing of repair and the associated outcomes.
